# Predicting Illusory Contours Without Extracting Special Image Features

**DOI:** 10.3389/fncom.2018.00106

**Published:** 2019-01-18

**Authors:** Albert Yankelovich, Hedva Spitzer

**Affiliations:** ^1^Department of Biomedical Engineering, Faculty of Engineering, Tel Aviv University, Tel Aviv, Israel; ^2^Faculty of Engineering, School of Electrical Engineering, Tel Aviv University, Tel Aviv, Israel

**Keywords:** figure ground segregation, illusory contours, functional minimization, multiple perceptions, computational Gestalt

## Abstract

Boundary completion is one of the desired properties of a robust object boundary detection model, since in real-word images the object boundaries are commonly not fully and clearly seen. An extreme example of boundary completion occurs in images with illusory contours, where the visual system completes boundaries in locations without intensity gradient. Most illusory contour models extract special image features, such as L and T junctions, while the task is known to be a difficult issue in real-world images. The proposed model uses a functional optimization approach, in which a cost value is assigned to any boundary arrangement to find the arrangement with minimal cost. The functional accounts for basic object properties, such as alignment with the image, object boundary continuity, and boundary simplicity. The encoding of these properties in the functional does not require special features extraction, since the alignment with the image only requires extraction of the image edges. The boundary arrangement is represented by a border ownership map, holding object boundary segments in discrete locations and directions. The model finds multiple possible image interpretations, which are ranked according to the probability that they are supposed to be perceived. This is achieved by using a novel approach to represent the different image interpretations by multiple functional local minima. The model is successfully applied to objects with real and illusory contours. In the case of Kanizsa illusion the model predicts both illusory and real (pacman) image interpretations. The model is a proof of concept and is currently restricted to synthetic gray-scale images with solid regions.

## Introduction

**Figure 1:**
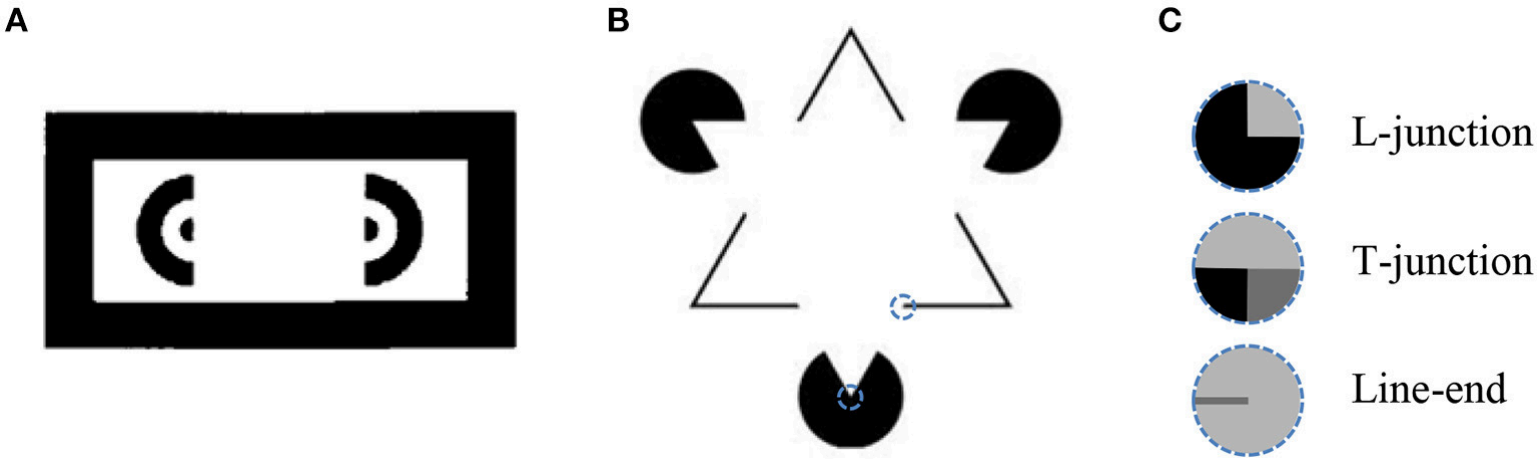
**(A)** An illusory rectangle is perceived between the halves of the donut (Schumann, 1900). **(B)** An illusory triangle that occludes three solid circles is perceived (Kanizsa, 1955). **(C)** Some special image features that are found at specific points in input image and are used for generating illusory contours, see text. The dashed line circles represent a piece of image and the special feature location is at the center of the circle. Example of L-junction and line-end are shown in Kanizsa triangle B. The features location is indicated by dash line circle.

An important and non-trivial task in process of image understanding is the detection of *object boundaries*, also termed *figure-ground segregation* or *image segmentation*. This task is especially difficult in conditions where the object boundary is not fully visible. The human visual system, in many cases, is able to construct the whole object boundary (Kanizsa, 1955). An extreme example of such a completion is demonstrated by illusory contours (Figures 1A,B), where the visual system “creates” object boundaries in locations without any intensity gradient (Schumann, 1900; Ehrenstein, 1925; Kanizsa, 1955; Gregory, 1972; Kennedy and Lee, 1976; Day and Jory, 1980; Prazdny, 1983; Bradley, 1987; Kennedy, 1988).

While numerous models for performing image segmentation have been reported (Leclerc, 1989; Nitzberg and Mumford, 1990; Pal and Pal, 1993), relatively few are designed to incorporate illusory contours. Most of the models are capable of generating illusory contours by extracting special image features, such as L-junctions, T-junctions, and line-ends (Figure 1C), and using them as key-points to create the illusory contours (Finkel and Edelman, 1989; Guy and Medioni, 1993; Williams and Hanson, 1994; Gove et al., 1995; Williams and Jacobs, 1995; review: Lesher, 1995; Kumaran et al., 1996; Heitger et al., 1998; Kogo et al., 2002; Ron and Spitzer, 2011). This approach is supported by psychophysical evidence that the existence of special image features are required for illusory contours to emerge (Rubin, 2001). Many of these models exploit neurophysiological knowledge about neuronal mechanisms of the visual system. For example, in the model of Heitger et al. (1998), the responses of *end stopped cells* that detect L-junctions and line-ends are grouped and added to the responses of *simple cells*, which detect image edges (image intensity gradient) to produce the illusory contour.

The special features extraction is a difficult task in real world images, since in order to decide which junctions are significant relative to others, the structure of the scene in the image needs to be understood (Nitzberg and Mumford, 1990). In addition, the fact that only a small fraction of the image is exploited for special feature extraction (image region around the special feature point) makes this approach less robust.

A widely accepted explanation of illusory contours is the perception of relative depth, where the illusory contour represents the boundary of an object located at an other depth than the region around it (Kanizsa, 1955; Coren, 1972; Gregory, 1972; Lesher, 1995). According to this point of view, the illusory contours are just regular object boundaries, with the object intensity being the same as that of the background. The object with the illusory contour is revealed by the objects that are being occluded behind it, as in Figure 1B. The special image features, such as L-junctions and line ends, can provide a clue for object occlusion. Extracting special features, however, means making a specific effort for illusory contours detection. In this case the illusory contours are not treated as the regular contours. We prefer not to extract special features and to use instead a common way to detect both real and illusory object boundaries. Detection of illusory contours without using special image features is very challenging, since it requires the prediction of contours *ex nihilo*, without using the *occlusion clues*.

An approach that has the potential of not extracting special image features is the functional optimization, used by some boundary detection models capable of generating illusory contours (Kass et al., 1988; Madarasmi et al., 1994; Williams and Hanson, 1994; Geiger et al., 1996; Saund, 1999; Gao et al., 2007). The functional is used to give a score for each contour configuration, and the final contours are not “constructed” by the model, but rather “come out” as the minimizer of the functional. Special features extraction is not necessarily required in these models, since the demand that the resultant boundaries will match the input image can be expressed in the functional without the special features extraction. An additional significant advantage of functional optimization approach is that giving a preference score to a given contour configuration is much simpler than constructing the correct contour configuration. The optimization approach is a computational realization of the Gestalt psychology (Koffka, 1935), since it derives the contours from some contour configuration preference rules (“grouping rules” in Gestalt psychology). By this it accounts for both real and illusory contours based on a general unified approach.

Kass et al. (1988) applied *snakes* algorithm of energy minimizing splines to track image edges. The continuity and elasticity properties of the snakes enable the illusory contours to emerge. This model indeed does not extract special image features, however, it is not fully automatic, since user interaction is required to draw the initial contour. One might argue that some automatic initial contours such as small circles matrix can be used, however in this case illusory contours will be extracted even for images that actually lack them. For example, the model will predict illusory contours for a Kanizsa illusion configuration with solid circle inducing elements, although in this case the illusory contour is not perceived. Currently there is no fully automatic boundary detection model that does not require special features extraction for illusory contours generation.

The proposed model is a proof of concept and is restricted to gray scale images with solid non-textured regions and without lines. The stress in the model is not on the way used to encode the Gestalt rules, nor on the rules themselves, but on the mere possibility by predicting real and illusory boundaries based solely on general boundary formation rules.

## Methods

### Model Rational

**Figure 2:**
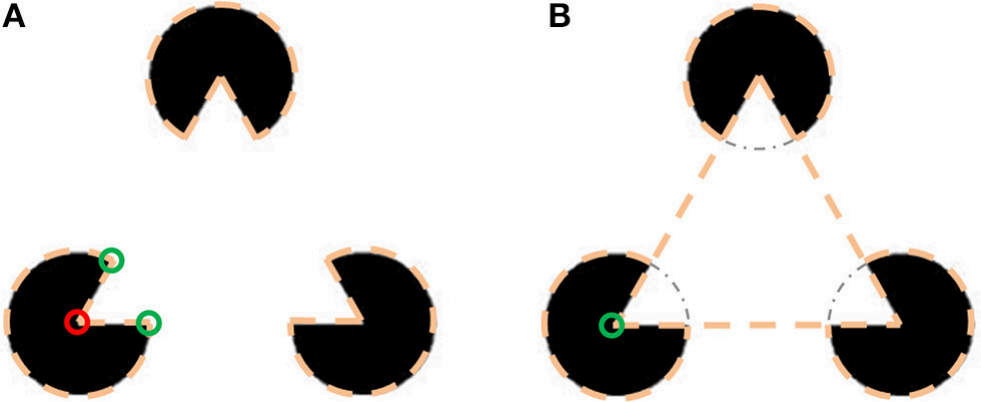
Illustration of two interpretations of the Kanizsa triangle. **(A)** Three objects having shape of a pacman. The dashed lines represent the perceived objects. Boundary bends are marked by small circles, red for concave and green for convex. Only one of the pacmans is marked to avoid burden. **(B)** Illusory triangle occluding three circular objects. The circular objects are perceived as being occluded by the triangular object. The dash-dot arc marks the circular object part behind the triangular object.

The basic idea of the model was inspired by the assumption that object detection is one of the intelligent tasks performed by the visual system. This task uses a set of simple assumptions, based on our natural perception of an object's appearance, to provide the most reasonable “explanation” of what is presented in the image. With several possible perceptions of what we see, a critical question is what makes us prefer one perception over another? Especially we are interested to reveal the reason for perception of illusory contours. As an example, let us consider the Kanizsa triangle (Kanizsa, 1955) in Figure 2 and examine the factors responsible for the perception of an illusory contour in this case.

The perception in Figure 2A is that of three “pacman” objects and the perception in Figure 2B is of a triangular object above three circular objects. In the pacman perception the boundary of each pacman has three corners (or bends)–two convex and one concave. On the other hand, in the triangle perception instead of three bends per pacman there is only one, since the circle is perceived as continuing under the triangle. Moreover, the concave bend in the circle center is replaced by a convex bend of the triangle vertex. The conclusion is that in the illusory interpretation the object's boundary is less bent and the bends are more convex. Both criteria can be derived from preference of simplest description (van Tuijl, 1975). The preference for convex bends also explains why in the image containing a square (Figure 7D), we perceive a square object more readily than a square hole.

Although the functional optimization approach enables us to avoid special features extraction, it has the drawback of having a tremendous search space of the possible solutions. To overcome this issue, we use an “economic” boundary representation called a *border ownership map*, holding boundary segments in discrete locations and discrete directions. Our representation is inspired by the neural findings of Zhou et al. (2000) who discovered V1 visual cortical cells that respond to an edge only when the object is located on one of the edge sides. This ability was already termed *border ownership* by Nakayama and Shimojo (1990). Using the border ownership map makes the free variable of the problem much smaller than using, for example, contour parametrization.

An additional difficulty is that the functional that accounts for several object boundary properties and depends on many variables has a large number of local minima. To overcome this, the functional was smoothed and the functional minimizers were found by gradual relaxation technique (Lee, 1995). This reduces the number of minima by smoothing out the shallow minima and finding only prominent stable minima.

In the proposed model we define a functional that accounts for basic object properties, such as boundary continuity and convexity, and demands the object boundaries to match the input image. The object boundaries are found as the minimizer of the functional. The illusory contours are predicted in same way as the real contours, by being the most probable object boundaries matching the input image. This is the first time that the perception of illusory contours from a general object boundary detection task is shown computationally.

The minimizers of the functional are compared to the expected perception, known from psychophysical evidence. Due to the suggestion that the visual system is actually finding the best solution to object formation rules, we are not necessarily obliged to use the mechanisms of the visual system (which are also not fully known), to find that solution. It has to be noted that in spite of this the model exploits some of the physiological knowledge of low-level mechanisms of the visual system, such as simplification of visual cell receptive fields that perform edge detection [section Border ownership at image edges (*F*^A^)], logical “and” operation (Appendix section 1.2) and cell response grouping (Appendix section 1.1). In addition, the model includes the crucial component of the border ownership map, section Boundary Representation.

Using functional minimization in the model has an additional important benefit. Usually, there are several possible object configurations that can explain a single image (Figure 2). Multiple image interpretations are present even in a simplest image of a white square on black background (Figure 7D). This image can be interpreted as a white square object over a black background, or as a black frame with a square hole through which a white background is seen. The illusory Kanizsa triangle (Figure 1B), also has several possible interpretations. The most prominent is the illusory interpretation of a white triangle occluding three black solid circles and a black boundary triangle (Ringach and Shapley, 1996). An additional easily perceived interpretation does not include an illusory triangle, but consists of three cut-out circles, “pacmans”, and three V-shaped figures. For real-world images there may be numerous plausible configurations of objects. The desired interpretation may be chosen, for example, by applying a higher level knowledge, like object recognition. The ability to predict multiple possible perceptions of the image is therefore a desired property of a robust boundary detection model. The multiple possible image interpretations, that are described above, are represented in our model by multiple minima of the functional.

### Model Overview

The model consists of four main parts:

1. Encoding of object boundaries.
2. The cost functional, specifying a cost value for each object boundaries configuration.
3. A method to identify object boundaries with minimal cost.
4. A method of finding multiple functional minima, corresponding to different image perceptions.

The main challenge of identifying illusory contours as a solution of a minimization problem is occupying the huge size of the solutions space. We attacked this problem by choosing a compact boundaries representation method and by applying various types of smoothing to the functional, in order to reduce the number of local minima. The smoothing leaves only the stable minima. A method was invented to find different local minima of the cost functional, section Finding Multiple Local Minima. Each local minimum corresponds to a possible image interpretation, with a lower cost for a more probable (pop-out) interpretation.

The variables notation below is that the subscript of a variable describes the discrete coordinate on which this variable is measured. For example, *f*_*xy*_ is a filter intensity at coordinate (*x, y*), for integer *x* and *y*. There are no continuous coordinates in the model. We omit the comma between the coordinates for brevity. The superscript of a variable is part of the variable name. For example, σ^*X*^ is a constant. In the following we describe the model parts in more detail.

### Boundary Representation

**Figure 3:**
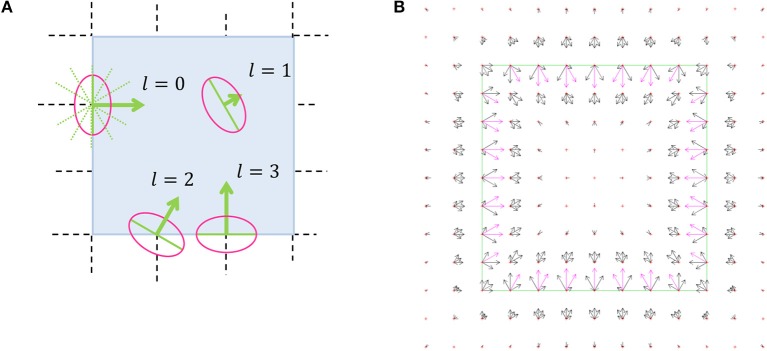
Border ownership map illustration. **(A)** Schematic illustration. The bold arrows are the border ownership vectors, while the vector length indicates the edge strength and the vector direction indicates the edge direction. The length of the ownership vector represents the probability that there is an object edge that passes through the vector origin in orientation perpendicular to the vector. The object is located on the side pointed by the border ownership vector. *l* is the discrete direction index, ranging from 0 to (*L*−1). Here and in all other border ownership maps *L* = 12. At each coordinate there is a border ownership vector for all possible directions, although for sake of clarity only some of the vectors are shown here. The dashed lines represent the discrete grid over the image of a square (the dark area). The dotted diagonal lines going out of the origin of *l* = 0 vector which show all the possible discrete directions. The ellipse around the vector origin illustrates the area in which the object edge is represented by the border ownership vector that is relevant. This resembles the receptive field of a V1 neuron (Hubel and Wiesel, 1959). **(B)** The output of border ownership map of the model for an input of a square object image. The border ownership vectors point inside the square (solid line). The vector with the greatest length at a point is directed perpendicular to the square edge.

The *border ownership map* (Figure 3A), represents the probability that an object edge passes through a discrete coordinate in some discrete direction. The orientation of the object edge is perpendicular to the discrete direction, and the object resides on the side that is pointed by the pointed direction. As an example, Figure 3B represents the border ownership map of a square object. At each discrete coordinate, the border ownership is specified for a discrete set of equally distributed *L* orientations (Figure 3A). Note that for opposite directions there are two different border ownership values. The border ownership is not strictly a probability value. Only the relative values of border ownership are important. We choose to interpret positive and negative values of border ownership in the same way, since in the minimization process additional effort is required to avoid negative values. To achieve this interpretation, the border ownership always appears squared in the functional.

### Cost Functional

The functional that depends on the border ownership map is designed to measure to what extent the expected properties of the object boundaries configuration are followed. Each property is allocated a *cost functional component* and the overall cost functional is a weighed sum of all the components.

(1) $$\begin{array}{l} F\left(\vec{b}\right)=\alpha^{A}F^{A}\left(\vec{b}\right)+\alpha^{R}F^{R}\left(\vec{b}\right)+\alpha^{V}F^{V}\left(\vec{b}\right)\\ \;+\alpha^{N}F^{N}\left(\vec{b}\right)+\alpha^{C}F^{C}\left(\vec{b}\right)+\alpha^{E}F^{E}\left(\vec{b}\right) \end{array}$$

**Figure 4:**
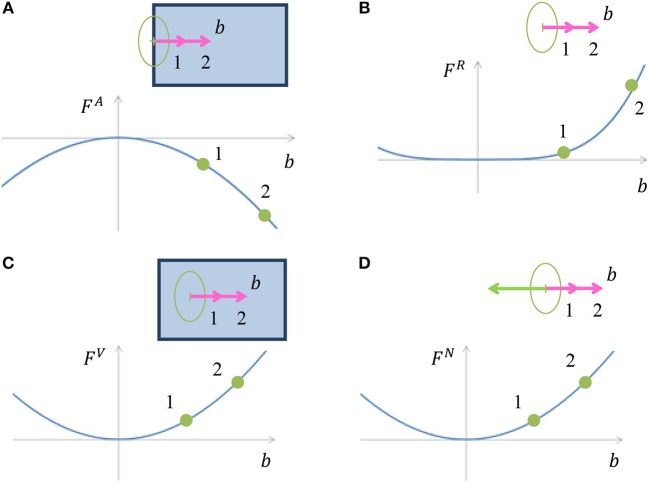
**(A)** Illustration of cost component *F*^*A*^, which is inducing border ownership in a direction perpendicular to the image edge. When a border ownership is of length denoted by 1 (pink arrow) in the image, the cost is as pointed in point 1 on the chart. For bigger border ownership denoted by 2 (pink arrow) in the image, the cost is as pointed in point 2, and is lower than the cost at point 1. **(B)** Illustration of border ownership limitation cost component *F*^*R*^. The cost increases with increasing the vector value of the border ownership in order to limit the infinite growth of the vector value, due to cost component *F*^*A*^. The polynomial degree of *F*^*A*^ in **(A)** is 2, while the polynomial degree of *F*^*R*^ is 4, which makes sure that the border ownership value will be limited. **(C)** Illustration of cost component *F*^*V*^, which gives penalty to border ownership in places with no edge in the image. The cost increases with increasing border ownership at a location with no edge in the image. **(D)** Cost component *F*^*N*^ discourages border ownership in opposite directions, since an object is expected to be only on one side of the edge.

**Figure 5:**
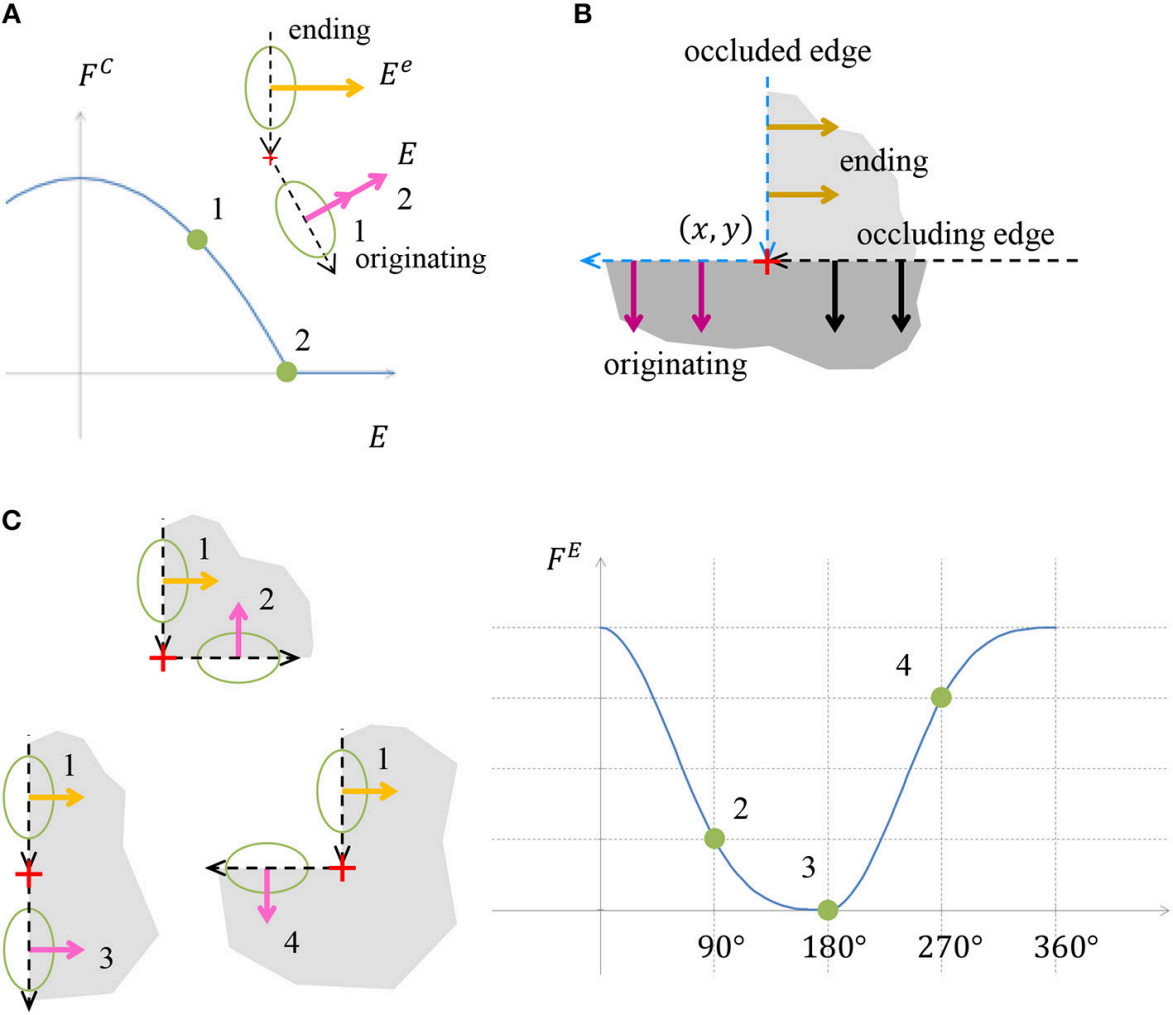
**(A)** Illustration of object edge continuity component *F*^*C*^. The value *E* is the strength of the object boundary edge originating from a specific location point. The chart presents the cost component value as a function of the originating edge strength *E*. The originating edge strength 1 is less than the strength of the ending edge, *E*^*e*^, hence a positive cost is assigned. Edge strength 2 is the same as the ending edge strength, thus the cost is zero. **(B)** Illustration of how the continuity is preserved in case of object boundary occlusion by additional object. The vertical edge is occluded below by an object with a horizontal edge. The occluding edge serves as the originating edge of the occluded ending edge. In this case no discontinuity is indicated by the continuity cost component ***F***^***C***^. **(C)** Illustration of the cost component accounting for object edge bending, *F*^*E*^. The object edge defined by vector marked 1 can continue by one of the edges marked by 2, 3, or 4. The costs for these three continuations are depicted in the chart. Note that the contribution of the convex continuation 2 is smaller than of the concave continuation 4, although both deviate by 90° from the straight continuation 3. The contribution of the straight continuation 3 is zero, since there is no boundary bending in this case.

Where *F*^*type*^ are the cost functional components that are dependent on the border ownership map

(2) $$\vec{b}={\left\{b_{xyl}\right\}}_{x,y,l}$$

and α^*type*^ are weight parameters. *x*, *y* are discrete coordinates and *l* is the discrete direction index. The first three components *F*^*A*^, *F*^*R*^ and *F*^*V*^ are responsible for appearance of border ownership at image edges. The other components are responsible for encoding the expected object boundary properties, and therefore depend only on the border ownership map and not on the input image. The component *F*^*N*^ is designed to make sure that the object is located only on one side of a boundary. *F*^*C*^ is responsible for object boundary continuity. *F*^*E*^ gives penalty for bending in the object boundary, while concave bends receive a greater penalty, section Model Rational. The cost components are visualized in Figures 4, 5 and are described in the following paragraph. Since the full definition of the components *F*^*C*^ and *F*^*E*^ is more complicated and occupy larger volume, their details are provided in Appendix in Supplementary Material.

#### Border Ownership at Image Edges (*F*^*A*^)

This chapter describes how border ownership is induced from image edges. In the case of an intensity edge in the input image with a specific orientation, the border ownership in the perpendicular direction is encouraged. Since we do not know on which side of the edge the object is situated, the border ownerships are encouraged in both directions which are perpendicular to the edge. The cost component sums multiplication of the border ownership *b*_*xyl*_ by the intensity of edge in the image in an orientation perpendicular to *l*, termed *A*_*xyl*_. This “encourages” border ownership perpendicular to the edge in input image (Figure 4A).

(3) $$F^{A}=\frac{1}{T}\sum_{x,y,l}^{}-A_{xyl}{}^{2}b_{xyl}{}^{2}$$

where

(4) $$A_{xyl}=I_{xy}*f_{xyl}^{A}$$

The operation marked by ^*^ is a discrete cross-correlation (or filtering), given by:

(5) $$I_{xy}*f_{xyl}^{A}=\sum_{x^{\prime },y^{\prime }}I_{\left(x+x^{\prime }\right)\left(y+y^{\prime }\right)}f_{x^{\prime }y^{\prime }l}^{A}$$

The filter $f_{xyl}^{A}$ detects an image edge at point (*x, y*) and orientation perpendicular to *l*. It is defined by rotation of function $f_{xy}^{A}$ by $2\pi \frac{l}{L}$.

(6) $$f_{xy}^{A}=\frac{1}{2\pi \sigma^{A}{}^{{}^{2}}}s\left(x\right)e^{-\frac{x^{2}+y^{2}}{2\sigma^{A}{}^{{}^{2}}}}$$

where function *s*(*x*) is a sign function, giving zero for values close to zero

(7) $$s\left(x\right)=\{\begin{array}{cc} 0, & \left|x\right|\leq 0.001\\ \frac{x}{\left|x\right|}, & else \end{array}$$

and σ^*A*^ is a constant. The constant *T* is used to normalize the cost to be per coordinate and orientation and is given by:

(8) $$T=I^{X}I^{Y}L$$

where *I*^*X*^ and *I*^*Y*^ are the width and height of the input image. The border ownership value *b*_*xyl*_ in (3) is squared in order to have same cost for positive and negative values of border ownership, section Boundary Representation.

#### Border Ownership Is Limited (*F*^*R*^)

If *F*^*A*^ was the only component of the functional, the border ownership at image edges would grow infinitely to make the cost lower. The following cost component is added to ensure that the value of border ownership is limited:

(9) $$F^{R}=\frac{1}{T}\sum_{x,y,l}^{}b_{xyl}{}^{4}$$

The reason for taking the border ownership to power 4 is to make *F*^*R*^ stronger than *F*^*A*^ at high border ownership values. The cost component *F*^*R*^ is illustrated in Figure 4B.

#### Suppress Border Ownership in the Absence of Image Edge (*F*^*V*^)

An illusory contour introduces border ownership also at places with no intensity gradient in the image. To avoid spurious illusory contours, this component adds a penalty for boundary ownership in places with no edge in the input image (Figure 4C).

(10) $$F^{V}=\frac{1}{T}\sum_{x,y,l}^{}\frac{\varepsilon^{V}}{A_{xyl}{}^{2}+\varepsilon^{V}}b_{xyl}{}^{2}$$

where ε^*V*^ is a small constant and *A*_*xyl*_ is intensity of edge in the image (4), used in component *F*^*A*^. Note that the equation and the rational of *F*^*A*^ (3) and *F*^*V*^ are similar, but have opposite trends, such that a large edge leads to lower cost, while a small edge causes a higher cost. The only functional components that depend on input image are *F*^*A*^ and *F*^*V*^. They depend only on image edges and not on special image features, as required in previous models, section Introduction.

#### Object on One Side (*F*^*N*^)

The model assumes that the object usually resides on only one side of an edge. Hence, if there is border ownership in a specific direction, the border ownership in the opposite direction is discouraged (Figure 4D). If there is a significant border ownership in direction *l*, border ownership in opposite direction $l+\frac{L}{2}$ is not expected, section Boundary Representation. Border ownership is also not expected in directions close to $l+\frac{L}{2}$, therefore, we add a cost for border ownership vectors with deviation *m* from $l+\frac{L}{2}$. We also consider border ownership in spatial vicinity to the border ownership vector origin (*x, y*) by filtering the border ownership map in space. The filtered border ownership map is termed $B_{xyl}^{N}$.

(11) $$F^{N}=\frac{1}{TT^{N}}\sum_{x,y,l}\sum_{m=-\left(\frac{L}{4}-1\right)}^{\frac{L}{4}-1}\cos^{2}\left(2\pi \frac{m}{L}\right)B_{xyl}^{N}B_{xy\left(l+\frac{L}{2}+m\right)}^{N}$$

where

(12) $$B_{xyl}^{N}=b_{xyl}{}^{2}*f_{xy}^{N}$$

and

(13) $$f_{xy}^{N}=\frac{1}{2\pi \sigma^{N^{2}}}e^{-\frac{x^{2}+y^{2}}{2\sigma^{N}{}^{2}}}$$

For a larger deviation *m*, the cost increase should be smaller, thus a weight factor cos^2^ $\left(2\pi \frac{m}{L}\right)$ is added accordingly. The maximum deviation considered is $\frac{L}{4}-1$, since this is the maximum angle which is less than $\frac{\pi }{2}$. The term *T*^*N*^ (11) is used to normalize the contributions from all deviations and is given by

(14) $$T^{N}=\sum_{m=-\left(\frac{L}{4}-1\right)}^{\frac{L}{4}-1}\cos^{2}\left(2\pi \frac{m}{L}\right)$$

#### Object Boundary Continuity (*F*^*C*^)

One of the basic properties of an object is the continuity of its boundary, thus the boundary is not expected to end abruptly, unless it is occluded by the boundary of another object. To encourage object boundary continuity, we require that when an object edge ends at a coordinate, there should be an object edge originating from the same coordinate (Figure 5A). The occluding object edge plays the role of the originating edge to the occluded object ending edge, in case of occlusion (Figure 5B). The main innovation of the model is the mere possibility to predict illusory contours without special features extraction, following the functional optimization approach. Since the full details of this component are quite lengthy and the exact functional definition is not the main aim of the model, this component details are provided in Appendix section 1.1.

#### Object Boundary Bending (*F*^*E*^)

We concluded in section Model Rational that the preferred perception is the one with fewer bends, and if there are bends, then convex bends are preferable. Taking this preference into account, we will assign a positive cost for bends in the object boundary, with an increased penalty for concave bends (Figure 5C). The details of this component are also lengthy, hence they are provided in Appendix section 1.2.

### Cost Functional Smoothing

The cost functional (1), accounting for several object boundary properties and depending on many variables, has a large number of local minima, while not all of them represent expected image interpretations. The problem is then how to “get rid” of these redundant local minima. We assume that the redundant local minima are shallower than desirable ones. To avoid trapping in shallow local minima, four types of smoothing methods are applied, as described in the following sections.

#### Border Ownership Map Smoothing in Angle and Space

To make the cost functional less sensitive to small changes in border ownership, the border ownership map $\vec{b}$ is smoothed in angle and space. The result ${\vec{b}}^{S}$ is used as input to the cost functional (1).

(15) $$b_{xyl}^{S}=\left[\sum_{j=-\left(\frac{L}{2}-1\right)\;}^{\frac{L}{2}}b_{xy\left(l+j\right)}f_{j}^{SA}\right]*f_{xy}^{SX}$$

where $f_{j}^{SA}$ and $f_{xy}^{SX}$ are Gaussians in angle (A) and space (X) coordinates, respectively:

(16) $$f_{j}^{SA}=\frac{1}{\beta^{SA}}e^{-\frac{j^{2}}{2\sigma^{SA}{}^{{}^{2}}}}$$

(17) $$f_{xy}^{SX}=\frac{1}{2\pi \sigma^{SX}{}^{{}^{2}}}e^{-\frac{x^{2}+y^{2}}{2\sigma^{SX}{}^{{}^{2}}}}$$

with σ^*SA*^ and σ^*SX*^ constants, and β^*SA*^ is a normalization constant:

(18) $$\beta^{SA}=\sum_{m=-\left(\frac{L}{2}-1\right)}^{\frac{L}{2}}e^{-\frac{m^{2}}{2\sigma^{SA}{}^{{}^{2}}}}$$

#### Spatial Filters Smoothing

The cost functional calculation uses various spatial filters. To make the cost smoother and less dependent on the discrete grid step, we sum up the cost components on multiple spatial scales.

(19) $$G^{type}=\frac{1}{N}\sum_{n=0}^{N-1}F_{n}^{type}$$

where *N* is the number of scales and $F_{n}^{type}$ is the same as *F*^*type*^ (1), except that it uses spatial filters derived by scaling the original filters by factor

(20) $$\mu^{n}$$

where μ > 1 is a scaling constant. The smoothed components *G*^*type*^ (17) are used in the functional instead of the components *F*^*type*^ (1).

#### Ramp Function Smoothing

The ramp function

(21) $$r\left(x\right)=\{\begin{array}{cc} 0,\; & x\leq 0\\ x, & x>0 \end{array}$$

is used in components *F*^*C*^ and *F*^*E*^ to account for positive and not negative values. There are two benefits in smoothing the ramp function *r*(*x*). The first is that the smoothed function is differentiable at *x* = 0 and the second is that the cost functional also becomes smoother, which reduces the number of local minima. The smoothed function is obtained by filtering *r*(*x*) through a Gaussian function:

(22) $$\frac{1}{\sqrt{2\pi \sigma^{RP}{}^{{}^{2}}}}e^{-\frac{x^{2}}{2\sigma^{RP}{}^{{}^{2}}}}$$

Where σ^*RP*^ is a constant.

#### Gradual Relaxation-Find the Minimum at Coarse to Fine Scale

In order to avoid trapping into shallow local minima, the minimum is found first on a coarse and then at a finer scale, a method called gradual relaxation (Lee, 1995). This is done by first finding the minimum of the functional on a broad scale. Then, the border ownership found is used as the initial point for finding the minimum on a finer scale. This process is repeated until the desired detailed scale is reached. The details of this process are as follows. A *scale parameter s* is initially set to *s*^0^ > 0. To proceed to a more detailed scale, the scale parameter *s* is multiplied by constant *s*^*R*^ with 0 < *s*^*R*^ < 1. The process is finished when the desired resolution of *s*^*M*^ is reached. For the scale *s*^*M*^ the smoothed functional is close to the functional without smoothing. The scale parameter *s* influences the model as follows.

The border ownership smoothing scale σ^*SX*^ (17) is multiplied by:

(23) $$s^{B0}+s^{BS}s$$

where *s*^*B*0^ and *s*^*BS*^ are constants. The scale μ^*n*^ (20) of spatial filters smoothing, is multiplied by:

(24) $$s^{X0}+s^{XS}s$$

where *s*^*X*0^ and *s*^*XS*^ are constants. The width of Gaussian (22) used for the ramp function smoothing is multiplied by:

(25) $$s^{R0}+s^{RS}s$$

where *s*^*R*0^, *s*^*RS*^ are constants.

### Finding the Local Minimum

The search for a minimum starts from a random border ownership map ${\vec{b}}^{R}$, with component values selected from a uniform random distribution, in the range [0.01, 0.02]. The reason for starting with a random border ownership rather than a zero vector is to avoid being trapped in a saddle point. For each scale parameter *s*, section Gradual Relaxation-Find the Minimum at Coarse to Fine Scale, the method used to search for the local minimum is a variant of a gradient descent (Curry, 1944). Suppose that at gradient descent iteration *i*, the current border ownership map is ${\vec{b}}^{i}$. We find the derivative of cost functional at ${\vec{b}}^{i}$ with respect to each border ownership component *b*_*xyl*_:

(26) $$\vec{D}=\frac{\partial F}{\partial \vec{b}}\left({\vec{b}}^{i}\right)={\left\{\frac{\partial F}{\partial b_{xyl}}\left({\vec{b}}^{i}\right)\right\}}_{x,y,l}$$

$\vec{D}$ is a matrix pointing in the direction of the greatest increase of *F* (1). To move toward the minimum of *F*, we need to move in the opposite direction $-\vec{D}$. The functional *F* near the minimum is roughly second order, see Appendix section 1.3. Based on this, we approximate the values of *F* along $-\vec{D}$ by a parabola and move to its minimum. The details of this process are specified in Appendix section 1.3.

### Finding Multiple Local Minima

The multiple local minima of the cost functional correspond to different interpretations of the image, section Introduction. Although there are several well established methods for finding a single minimum of a functional, there are relatively few studies on how to find multiple minima. The main question is how to escape from the first local minimum, in which the minimization process stopped. We attack this problem by positioning a “repulsive particle” in the location of the first local minimum. Here by location we mean the border ownership map of the minimum. The repulsive particle acts like an electric charge that repulses the border ownership map that is being searched and prevents it from coming too close to the repulsive particle location. This is achieved by adding to the cost functional (1) a component that increases for border ownership maps that are close to the first local minimum. This component is described in details in Appendix section 1.4, and it resembles an electric potential. The process of finding multiple local minima is performed as follows.

**Figure 6:**
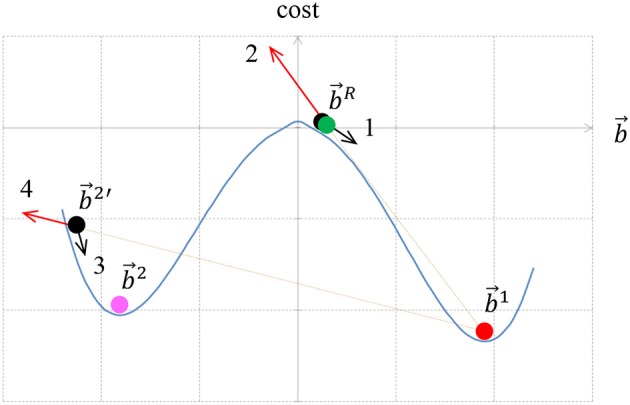
The method for finding multiple local minima of a functional. We start from a random particle ${\vec{b}}^{R}$ (green) and reach a minimum at ${\vec{b}}^{1}$ (red). The force that is responsible for moving the particle from ${\vec{b}}^{R}$ is caused by the cost functional (arrow 1 black). The particle ${\vec{b}}^{1}$ is then replaced by an immovable repulsive particle at the same location. When the process is restarted with a new particle from ${\vec{b}}^{R}$ (black), the repulsive particle at ${\vec{b}}^{1}$ pushes the particle at ${\vec{b}}^{R}$ (red arrow 2) toward a new minimum ${\vec{b}}^{2}$. However, due to the repulsive force (red arrow 4) the particle is pushed beyond the new minimum ${\vec{b}}^{2}$, and arrives at location ${\vec{b}}^{2^{\prime }}$ (black). To find a local minimum, which is not influenced by the repulsive particle, the repulsive particle at ${\vec{b}}^{1}$ is removed and a new minimum search starts from ${\vec{b}}^{2^{\prime }}$. This particle is pushed to a new minimum at ${\vec{b}}^{2}$ (magenta) by the cost functional force (arrow 3 black).

The gradient descent starts from some random border ownership ${\vec{b}}^{R}$, section Finding the Local Minimum, to obtain a local minimum for border ownership ${\vec{b}}^{1}$, (Figure 6). To find additional local minimum we place a repulsive particle at the ${\vec{b}}^{1}$ position (red ${\vec{b}}^{1}$ in Figure 6) and reinitiate the search for new local minimum from ${\vec{b}}^{R}$. Suppose that now the new local minimum is ${\vec{b}}^{2^{\prime }}$ (magenta ${\vec{b}}^{2^{\prime }}$). The repulsive particle at ${\vec{b}}^{1}$ causes ${\vec{b}}^{2^{\prime }}$ to be pulled out further from the actual local minimum of the cost functional. To find the actual local minimum, we start a new search for the minimum of the functional without repulsive particle component from location ${\vec{b}}^{2^{\prime }}$. Suppose the search reached the minimum for ${\vec{b}}^{2}$.

If ${\vec{b}}^{2}$ is sufficiently far from ${\vec{b}}^{1}$, then ${\vec{b}}^{2}$ is added as a new interpretation and a repulsive particle is added at ${\vec{b}}^{2}$. To measure how close ${\vec{b}}^{1}$ is to ${\vec{b}}^{2}$, the following simple distance measure is used:

(27) $$\frac{1}{T}\sum_{x,y,l}^{}\left|b_{xyl}^{1}{}^{2}-b_{xyl}^{2}{}^{2}\right|$$

where *T* is defined in (8). If this distance is above a specific threshold level *d*^*T*^, the particles are considered different. If ${\vec{b}}^{2}$ is close to ${\vec{b}}^{1}$ (27), then the optimization is trapped into a local minimum that has been already identified. Since the search was trapped twice in the same local minimum, we try to increase the force of the repulsive particle. This is achieved by multiplying the repulsive term by a constant factor τ>1. In order to avoid the same location ${\vec{b}}^{2^{\prime }}$ again, an additional repulsive particle is added at the ${\vec{b}}^{2^{\prime }}$ location, and the search for the minimum is repeated from a start point at ${\vec{b}}^{R}$ (Figure 6). After finding this minimum we perform a new search, but without the repulsive particle component, in order to find the actual local minimum of the original functional. If a new particle is found, then the new particle is added as additional interpretation. The repulsive force is returned to its initial strength (without multiplication by τ) and a search for a new particle is performed. If, on the other hand, no new particle is found, the repulsive force factor is multiplied again by τ. The repulsive force multiplication factor is increased until a maximum factor τ^max^ is reached. If even for the maximum multiplication factor no new particle is found, then the process of finding multiple local minima is stopped.

### Retrieving Object Shape by Contour Evolution

At this stage, the output of the model is a border ownership map (2) that assigns border ownership strength values to each discrete location and direction. To show that the actual object shape can be easily and automatically retrieved from the border ownership map, we designed a simple contour evolution algorithm that finds the top-most object in the scene. The contour evolution method finds a contour which maximizes a given functional that depends on the contour. The way to find the maximizing contour is by moving some initial contour toward the contour that brings the functional to maximum. In the level set approach, the contour is represented by the intersection of a two dimensional function ψ with x-y plane, that is by the zero-level of the function ψ. The contour motion is described and performed in terms of the function ψ. For further details see Osher and Sethian (1988).

We start with a simple small object (e.g., circular contour) which is adjacent to the border ownership vector with the biggest value. The contour representing the object boundary is then moved to maximize the border ownership vectors having direction perpendicular to the contour. Following Malladi et al. (1995), the contour dynamics is defined by:

(28) $${\vec{C}}_{t}=\left(k-v\right)g\vec{N}$$

${\vec{C}}_{t}$ is the velocity of moving the contour $\vec{C}$. $\vec{N}$ is the contour normal vector, pointing toward the inner area of the object. The contour is moved in direction of the normal. The velocity magnitude is defined by (*k*−*v*)*g*, (28). This function is designed to cause the contour to grow until it reaches the highest value of border ownership vectors and to keep the contour as simple as possible. The term *k* is the contour curvature and the operation of including this term makes the contour tend to be as straight as possible. This is because a point with positive curvature, that is a convex point, the contour is “encouraged” to move inside, which decreases the curvature. For negative curvature the contour is “encouraged” to move outwards, decreasing the absolute curvature and again making the contour more straight. *v* is a constant called the balloon force, giving the contour the tendency to grow. The contour friction term *g* causes the contour to stop when it reaches a high value of border ownership vectors in the direction perpendicular to the contour. *g* is a threshold of another function *h*:

(29) $$g_{xy}=\{\begin{array}{cc} 0,\; & h_{xy}<g^{T}\\ h_{xy},\; & else \end{array}$$

(30) $$h_{xy}=\frac{1}{\left(1+\frac{q_{xy}}{R^{2}}\right)}$$

where *R* is a constant and *q*_*xy*_ measures the strength of the border ownership in a direction roughly perpendicular to the contour. *h*_*xy*_ is designed such that it will be small in locations where the value of the border ownership perpendicular to the contour is high. Since *h*_*xy*_ is small in this locations, *g*_*xy*_ will be zero and the contour evolution will stop. *q*_*xy*_ is given by:

(31) $$q_{xy}=\sum_{l=1}^{L}w_{l}b_{xyl}{}^{2}$$

The weighting factor *w*_*l*_ measures how close the direction *l* is to the direction of the contour normal:

(32) $$w_{l}=e^{-\frac{\beta_{l}{}^{2}}{2\sigma^{Q^{2}}}}$$

where σ^*Q*^ is a constant, and β_*l*_ is the angle between the direction of index *l* and the contour normal, pointing toward the inner area of the object:

(33) $$\beta_{l}=\cos^{-1}\left({\vec{\mu }}_{l}\cdot \vec{N}\right)$$

And ${\vec{u}}_{l}$ is the unit vector in direction of index *l*:

(34) $${\vec{u}}_{l}=\left(\cos\alpha_{l},\sin\alpha_{l}\right),\;\alpha_{l}=2\pi \frac{l}{L}$$

Further details of the approach in field of level set curve evolution can be supplied from Osher and Sethian (1988).

## Results

The model was tested on various simple synthetic gray scale images with non-textured regions. The same set of model parameters were used for all tests and stimuli. The parameters were chosen by trial and error.

**Figure 7:**
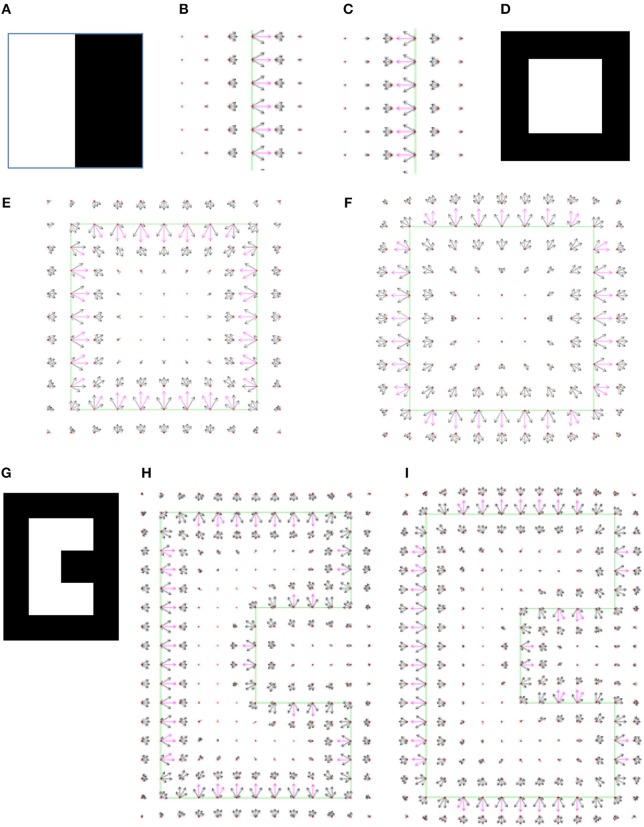
**(A)** The simplest input image, with size 20 × 20 pixels. **(B)** The border ownership map of the first model interpretation of the image **(A)**. The object is located on the right side of the edge that is between the white and the black area in the input image. In all border ownership maps shown in following figures, the edges in the input image are marked by green lines for reference. The border ownership vectors with a value above 80% of the maximum border ownership vector value in the current map are colored magenta. Other border ownership vectors are black. The small red crosses depict the discrete grid of the input image. Note that only part of the border ownership map is shown, in order to make the view clearer. **(C)** The second model interpretation represents an object on the left side of the boundary between the white and the black regions in the input image **(A)**. **(D)** Input image with white square 8 × 8 pixels on black background. **(E)** The first model interpretation of the image in **(D)** represents a white square object on black background. The interpretation has a lowest cost −117. **(F)** The second model interpretation of the image in **(D)** represents a black frame with a square hole through which a white background is seen. This interpretation has cost−102, higher than the first interpretation, meaning it is less probable. **(G)** Input image of a C-shaped object. A similar image was applied in the original study of border ownership neurons (Zhou et al., 2000). **(H)** The first model interpretation of image **(G)** represents a C-shaped object. **(I)** The second model interpretation of image **(G)** represents a C-shaped hole in a frame.

The first image contains two adjacent regions separated by a straight line (Figure 7A). Two local minima were found for this image, one corresponding to a black object on the right side over white background (Figure 7B), and the other one found relating to a white object on the left side over the black background (Figure 7C). Note that the two interpretations have equal cost −53.1, since there is no preference for the object to be on the right or on the left side.

The next tested image was a square (Figure 7D), also having two interpretations. The first interpretation was of a square object (Figure 7E), and the second interpretation was of a frame with a square hole (Figure 7F). The square object interpretation has cost −117, while the square hole in a frame interpretation has a higher cost −102. This is consistent with the fact that the square interpretation is perceived more readily than the square hole interpretation, section Model Rational. In all results the interpretations are presented ordered from lower to higher cost. The model behaves in the same manner for a larger square with size of 20 pixels (results are not shown). For a more complex image of an object with both convex and concave vertexes (Figure 7G), the model identifies two interpretations, the first corresponding to a C-shaped object (Figure 7H), and the second to a frame with a C-shaped hole (Figure 7I).

**Figure 8:**
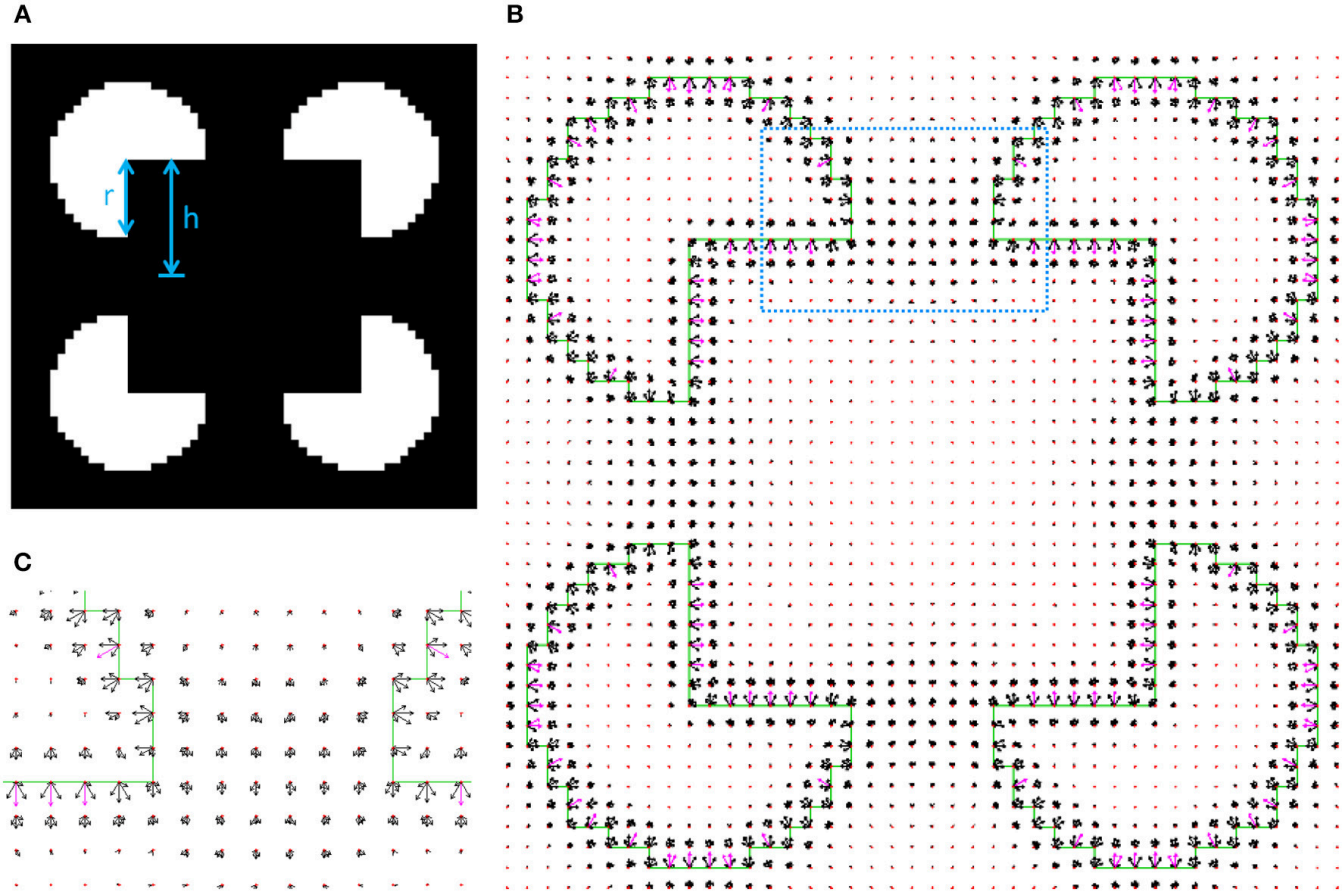
**(A)** The Kanizsa illusory square. In this image the support ratio is 0.67. (The support ratio is defined as *r*/*h*, where *r* is the radius of the pacman and *h* is half the size of the illusory square). **(B)** The first model interpretation representing the square object, with partially illusory contours, occluding four circular objects. **(C)** Zoom-in into illusory boundary region between two pacmans, marked with dotted square in **(B)**.

**Figure 9:**
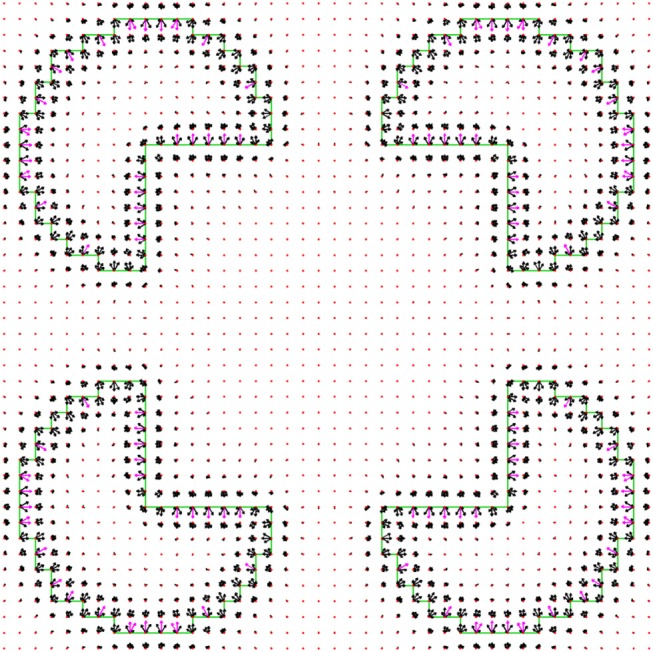
The second model interpretation of image in Figure 8A represents four pacman objects.

The main goal of the study was to show the possibility to detect objects with illusory contours without extracting special image features. To show this, the model was applied on Kanizsa squares with different sizes. One of the essential factors that determines the strength of the illusory contour is the ratio between the visible edge length and the total edge length, termed *support ratio* (Shipley and Kellman, 1992; Figure 8A). The illusory object is perceived when the support ratio values are close to 1. The model was tested on images corresponding to a broad range of support ratios. The first example is of a prominent illusory contour image (Figure 8A), with a relatively high support ratio of 0.67. The first interpretation, having the smallest cost −67.3, is the interpretation of an illusory square (Figures 8B,C). The second interpretation, having a higher cost −64.6, is of four pacemans (Figure 9). These two interpretations are consistent with our expectations from the model.

**Figure 10:**
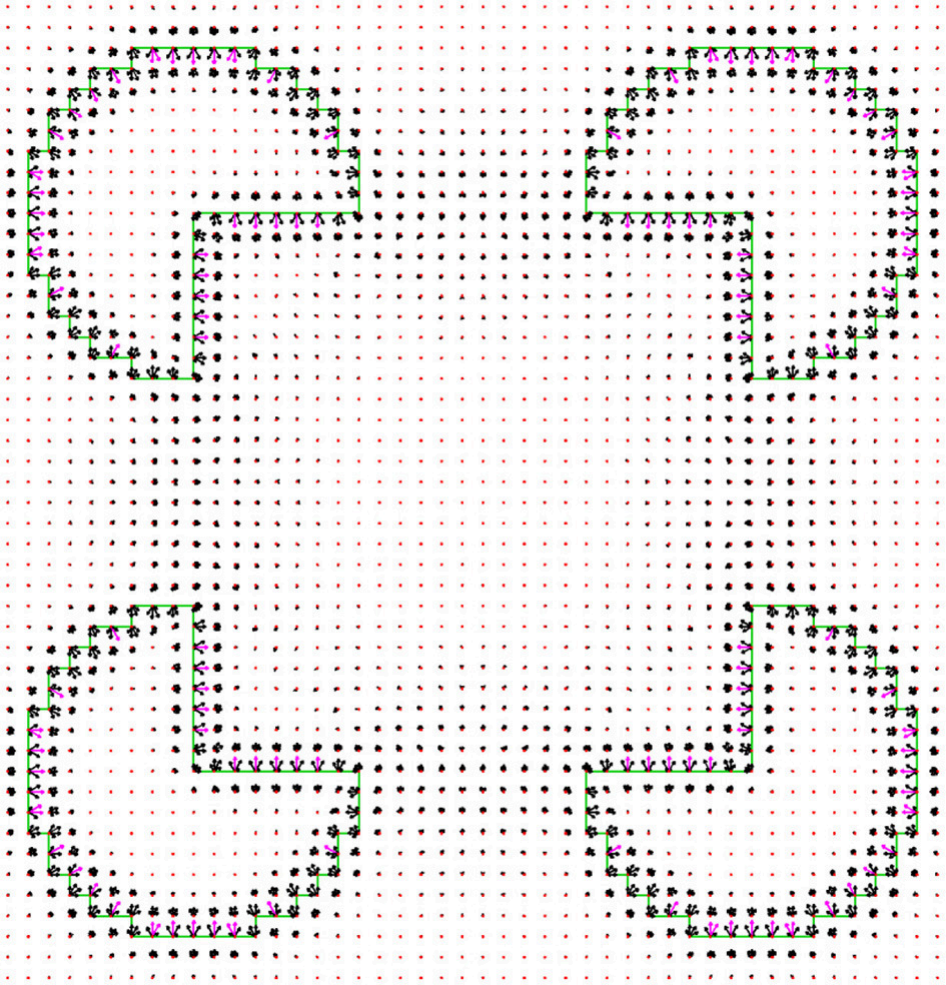
The first illusory interpretation of Kanizsa square image with support ratio 0.57.

Additional higher cost interpretations have been found, and are not presented here. The smallest support ratio for which the illusory square is still detected for this pacman radius is 0.57. Figure 10 shows the first interpretation for this support ratio. For a smaller support ratio of 0.53 the first interpretation is of four pacmans (the border ownership map is not shown, but has the same structure as the interpretation in Figure 9). For this support ratio there is no illusory interpretation at all, as expected.

**Figure 11:**
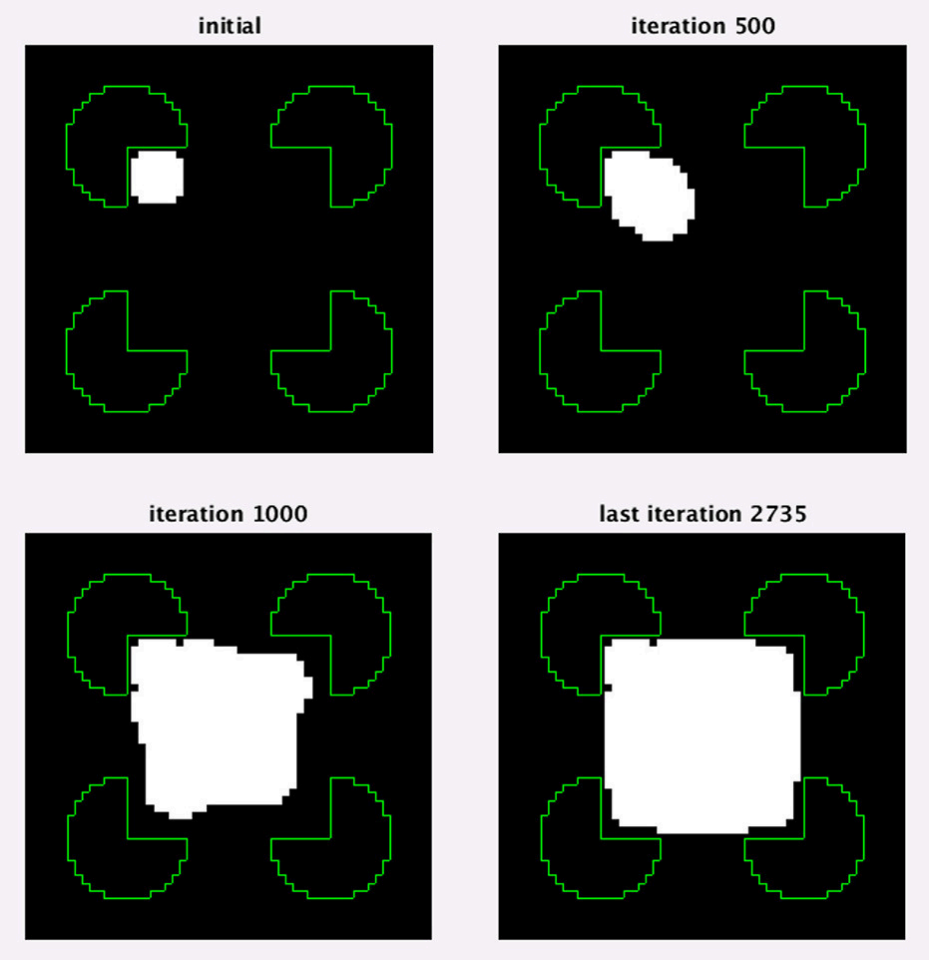
An optimization test showing that a square object can be determined from the border ownership map, found by the model. The object extraction is for the first interpretation of Kanizsa square with support ratio 0.57 (Figure 10). Four optimization stages at different number of iterations are shown. In the images, the white region is the object at the depicted iteration. The green lines show the input image edges, which are shown for reference.

To ensure that the illusory square border ownership map (Figure 10), can be interpreted as a square over four circles we applied a level set optimization method to extract the nearest object, section Retrieving object shape by contour evolution. The result of object extraction is shown in Figure 11. It shows detection of the square object with a partially illusory boundary.

## Discussion

The proposed model successfully extracts both real and illusory contours in various synthetic images (Figures 7–10). The model is generic and was not specifically designed to detect illusory contours, while special image features are not extracted. The illusory contour detection was achieved by introducing only simple desired object properties, and the illusory parts of the object boundary were generated as the most reasonable image “description” obtained by the functional minimization. The model shows the possibility to view the illusory contours as derived from general object detection task, performed by the visual system. Although this idea is not new (Gregory, 1972), this is the first time that the possibility to derive illusory contours from general object boundary detection task has been proved computationally.

Moreover, the multiple possible image perceptions were predicted here and ranked by perception probability. In case of the Kanizsa square illusion image, the most probable perception predicted by the model is of an illusory square (Figures 8B,C), and the second perception is of four pacman objects (Figure 9). Both predictions are consist with psychophysical findings (Rubin, 2001). Detecting different plausible solutions of a problem by finding multiple local minima of the functional is a novel approach.

There are numerous models that predict illusory contours in the Kanizsa square image (Williams and Hanson, 1994; Heitger et al., 1998; Kogo et al., 2002; Ron and Spitzer, 2011). The presented model approach, however, is essentially different from most of the models, since it is not oriented to detect illusory contours or locations of object occlusion. The model defines general preference rules of object boundaries and finds a stable minimizer to these rules. The illusory contours come out “by the way” as the minimizer of the problem. Since the essential approach of the model is the prediction of illusory contours based on general boundary detection approach, the model results cannot be compared to models that use specific mechanism of constructing illusory contours. The fact that the model does not use a general boundary detection approach is manifested by extraction of special image features.

Most of the existing models do extract special image features. For example, Madarasmi et al. (1994) use stochastic minimization of a functional to predict real and illusory contours of objects at different depth planes. The model is successfully applied to Kanizsa square illusion, where it detects both the illusory square and the overlapped inducer objects. The model, however, extracts special image features, namely L and T junctions, and only a single image interpretation is predicted. On the other hand, the model of Kass et al. (1988) detects real and illusory contours using energy minimizing splines. The model does not require special features extraction and both edge induced and line-end induced illusory contours are detected. However, the model is not fully automatic, since user interaction is required to draw the initial contour, section Introduction. In addition, only a single image interpretation is predicted in their model.

The functional optimization is usually used to obtain the best solution to a problem and only the global minimum is considered important (Figueiredo et al., 2003). Local minima are often considered to be disruptive and efforts are made to avoid them (Lee, 1995). The idea of a functional that has multiple minima is strongly related to the Gestalt psychology concept of Pragnantz: a *simple* and *stable* grouping (Koffka, 1935). Since the simplicity is measured by the cost functional, a local minimum of the functional indeed represents a simple and stable interpretation. Moreover, the values of the functional achieved at the different minima provide a general method, to compare the solutions at these minima. The multiple interpretations of the image are found in our model as the multiple stable minima of a functional. Thus, expressing multiple plausible solutions of a problem as multiple local minima of a functional is a new approach in the framework of functional optimization.

The method used to avoid minima that were already found in a functional section Finding multiple local minima, is related to the filled function method (Renpu, 1990), which has been used to find the global minimizer of a functional. In their method, an identified local minimum is replaced by a maximum in the functional. The main difference between the methods is the nature of the change in the function. The filled function depends on the functional in a complicated way, while in the proposed method the repulsive term is just added to the cost functional. In addition, our minimization is always initiated from the same point, while according to their method it requires trial over a set of directions, which is less efficient computationally.

The level set approach method section Retrieving object shape by contour evolution, can be used not only to find the top-most object boundary, but also the boundary of additional objects. To perform this, the initial small object should be placed adjacent to part of the boundary of the other object. This can enable us, for example, to complete the boundary of an occluded object.

The constants in the model were chosen by trial and error. Since the presented model proposes new a approach to the boundary detection task and contains a lot of complexity at this stage already, it is hard to also make it a fully robust model. Previous new conception models also did not supply a parameter sensitivity test at the first stage (Geiger et al., 1996). In any case, the same set of parameters were used for all experiments, hence we assume and experienced that the model is not very sensitive to parameter choice.

The proposed proof of concept model is restricted to gray scale images with solid non-textured regions and without lines. The model in its current version is not applicable yet for contour integration and detection of illusory lines such as defined by abutted gratings, since the model does not include components dealing with lines or texture. Dealing with such type of images will require us to extend the measure of “description length” in the functional (van Tuijl, 1975) to include textured regions. It is very interesting to compare the model to available psychophysical data, like classification images obtained from human participants (Murray et al., 2005), however this is currently out of scope of the presented preliminary model.

Future work is planned to develop a robust model for object detection in real-world images. For this purpose, the object boundary based approach of current model should probably be replaced by an area based approach. We expect that this change will make the model much simpler, since, for example, matching the image by regions does not require even extraction of edges in the image. This change can also enable us to account for region based effects in the Kanizsa illusion (Kanizsa, 1976; Grossberg and Mingolla, 1987; Spehar, 2000; Ron and Spitzer, 2011).

## Author Contributions

AY developed and tested the model. HS supervised the work, made contributions to the model and reviewed the paper.

### Conflict of Interest Statement

The authors declare that the research was conducted in the absence of any commercial or financial relationships that could be construed as a potential conflict of interest.
