# Supplementary material for: Predicting Illusory Contours Without Extracting Special Image Features
Source: Front Comput Neurosci. 2019 Jan 18;12:106. doi: 10.3389/fncom.2018.00106 (PMC6345704; doi:10.3389/fncom.2018.00106)
Supplement: Supplementary file 1 [file Data_Sheet_1.docx]

# APPENDIX

## Object boundary continuity cost component definition details ($\boldsymbol{F}^{\boldsymbol{C}}$)

To account for continuity of the object boundary we need a way to detect it in the border ownership map. Border ownership vectors along and perpendicular to a line are called *object* *ownership edge*, Figure 12A. The object ownership edge represents a part of object boundary. To encourage object boundary continuity, we require that when there is an *object ownership edge* that ends at a coordinate, there should be an object ownership edge that originates from the coordinate, Figure 12B. Below we refer to the *object ownership edge* simply as *object edge* or just *edge*. The requirement that there will be an originating edge for each ending edge should be satisfied also in the case of object occlusion. In this case the occluding object edge plays the role of the originating edge to the occluded object ending edge, Figure 5B.

$$\left( x,y \right)$$

**A**

$$\left( x,y \right)$$

ending

originating

**B**

FIGURE 12 | (A) Illustration of object ownership edge. The arrows are the border ownership vectors and the dashed line is the object ownership edge. (B) Illustration of object ownership edges ending in and originating from a coordinate.

First, we need a way of measuring the strength of the object edge. This is achieved by applying a filter that sums the border ownership in a direction that is perpendicular to that of the edge, as explained in the following. There are two opposite edge directions perpendicular to a given border ownership of direction $l$, Figure 13A. The object edge in direction $l+L/4$ is termed *positive* while in direction $l-L/4$ it is termed *negative*.

$$\left( x,y \right)$$

$$E_{xyl}^{P}$$

$$b_{x^{'}y^{'}l}$$

direction $l+\frac{L}{4}$

$$\left( x,y \right)$$

$$E_{xyl}^{N}$$

$$b_{x^{''}y^{''}l}$$

direction $l-\frac{L}{4}$

**A**

**B**

$$\left( x,y \right)$$

$$E_{xyl}^{P}$$

$$E_{xy\left( l+\frac{L}{2}-k \right)}^{N}$$

$$k$$

ending

originating

FIGURE 13 | (A) Illustration of object ownership edge strength. $E_{xyl}^{P}$ is the strength of the positive edge collecting border ownerhip vectors at direction $l$. The vectors are collected along a line that starts from the coordinate $\left( x,y \right)$ and has direction of $l+L/4$. $E_{xyl}^{N}$ is the strength of the negative edge also collecting border ownership vectors of direction $l$. The vectors are collected along a line that starts from the coordinate $\left( x,y \right)$ and has direction of $l-L/4$. (B) Illustration of ending and originating edges ($E_{xyl}^{P}$, $E_{xy\left( l+L/2-k \right)}^{N}$, respectively) with angle $k$ between them.

The strength of the object ownership edge is defined by

|  | $E_{xyl}^{P}={b_{xyl}}^{2}*f_{xyl}^{E}$ $E_{xyl}^{N}={b_{xyl}}^{2}*f_{xy\left( l+\frac{L}{2} \right)}^{E}$ | (35) |
| --- | --- | --- |
|  |  |  |

All direction calculations are modulo $L$. The filter $f_{xyl}^{E}$ is defined by rotating filter $f_{xy}^{E}$ by an angle of $2\pi l/L$. The filter $f_{xy}^{E}$ is given in radial coordinates by:

|  | $f_{r\theta}^{E}=\frac{1}{\beta}f_{r}^{E}f_{\theta}^{E}$ | (36) |
| --- | --- | --- |
|  |  |  |

where

|  | $f_{r}^{E}=-e^{-\frac{r^{2}}{2{\sigma^{C}}^{2}}}+e^{-\frac{r^{2}}{2{\sigma^{F}}^{2}}}$ | (37) |
| --- | --- | --- |
|  |  |  |
|  | $f_{\theta}^{E}=e^{-\frac{\left( \theta-\frac{\pi}{2} \right)^{2}}{2{\sigma^{T}}^{2}}}$ | (38) |
|  |  |  |

The angular function $f_{\theta}^{E}$ selects a sector of angles around $\pi/2$ and width $\sigma^{T}$. The radial function $f_{r}^{E}$ is basically a Gaussian function with width $\sigma^{F}$. Near the axes origin the angular function introduces a discontinuity so we exclude the origin by subtracting a Gaussian with smaller width $\sigma^{C}$. The normalization constant $\beta$ (36) is given by

|  | $\beta=\int_{0}^{\infty} \int_{0}^{2\pi} f_{r}^{B}f_{\theta}^{B}d\theta dr$ | (39) |
| --- | --- | --- |
|  |  |  |

To ensure that there is an originating edge for an ending edge, the strength of the originating edge has to be at least equal to the strength of the ending edge. For a given ending edge the sum of the strengths of all possible originating edges is subtracted from the ending edge strength. If the result is negative, no cost will be added but if the subtraction gives a positive result, meaning that the sum of the strengths of originating edges is lower than the ending edge, the cost will be increased, Figure 5A. Suppose first that the ending edge is of positive type and has strength of $E_{xyl}^{P}$ (35). Let $k$ be the angle between the ending and the originating edge. The originating edge is of negative type, Figure 13B. If the originating edge had same orientation as the ending edge ($k=0$), the originating edge strength would be $E_{xy\left( l+L/2 \right)}^{N}$, since the direction of border ownership vectors for the negative edge are opposite to those of the positive edge. Since the originating edge is rotated by $k$ relative to ending edge, the originating edge strength is $E_{xy\left( l+L/2-k \right)}^{N}$.

After we subtract the originating edge strengths we would like to assign zero cost for negative results and a positive cost for positive results. For this we use the ramp function $r\left( x \right)$ (21). There are two terms in the continuity cost component, one for positive edges continuation and the other for negative edges continuation.

|  | $F^{C}=\frac{1}{T}\sum_{x,y,l} \left[ r\left( E_{xyl}^{P}-\sum_{k=1}^{L-1} E_{xy\left( l+\frac{L}{2}-k \right)}^{N} \right)+r\left( E_{xyl}^{N}-\sum_{k=1}^{L-1} E_{xy\left( l+\frac{L}{2}+k \right)}^{P} \right) \right]$ | (40) |
| --- | --- | --- |
|  |  |  |

## Object boundary bending cost component definition details ($\boldsymbol{F}^{\boldsymbol{E}}$)

According to the model, there is a bend in the object boundary if there is an edge ending at a specific coordinate and an edge originating from that coordinate in a different orientation, Figure 12B. To check if there are both ending and originating edges we apply a multiplication operation of the edge strengths. The multiplication serves as a logical operation of AND. Thus, to account for a bending of angle $k$ we multiply the ending object ownership edge $E_{xyl}^{P}$ by the originating object ownership edge $E_{xy\left( l+\frac{L}{2}-k \right)}^{N}$, Figure 12B. To assign different cost for convex and concave bends, Section ‎2.4.6, the result of the AND operation is multiplied by a weight factor $e_{k}$ (depending on the bending angle $k$). The boundary bending cost component should be:

|  | $\tilde{F}^{E}=\sum_{x,y,l} \sum_{k=1}^{L-1} e_{k}E_{xyl}^{P}E_{xy\left( l+\frac{L}{2}-k \right)}^{N}$ | (41) |
| --- | --- | --- |
|  |  |  |

There is, however, a problem with this approach, as demonstrated in Figure 14.

2

3

1

FIGURE 14 | Illustration of the requirement to block an object ownership edge, when there is more than one possibility for continuation. Edge 1 is continuing by edge 2, hence there should be no cost incurred due to continuation by edge 3.

The object ownership edge 1, Figure 14, continuation with edge 3 produces a contribution to the cost component (41). However, there is actually no bending in the figure, since the continuation of edge 1 is by edge 2, and not by edge 3. The rationale for ignoring the continuation by edge 3 is that the angle of edge 2 relative to edge 1 ($180^{\circ}$) is smaller than the angle of edge 3 relative to edge 1 ($270^{\circ}$). The way to ignore the continuation via the greater relative angle is to “block” the greater relative angle edge by edges with a smaller relative angle. Blocking means making the edge with greater relative angle not to contribute to the edge bending cost. In the example above, the continuation of edge 1 with edge 3 is disregarded, since edge 3 is blocked by edge 2, which has smaller angle relative to edge 1. Consequently, the boundary bending cost component $F^{E}$ (1) becomes:

|  | $F^{E}=\frac{1}{TT^{E}}\sum_{x,y,l} \sum_{k=1}^{L-1} e_{k}E_{xylk}^{PB}E_{xy\left( l+\frac{L}{2}-k \right)k}^{NB}$ | (42) |
| --- | --- | --- |
|  |  |  |

where $E_{xylk}^{PB}$ is the result of “blocking” $E_{xyl}^{P}$ by edges having smaller angle relative to originating edge $E_{xy\left( l+\frac{L}{2}-k \right)}^{N}$. The blocked edge $E_{xylk}^{PB}$ is obtained by subtracting from $E_{xyl}^{P}$ the strengths of edges with directions within range $l-1,\ldots,l-k+1$, since these edges have smaller angle relative to the originating edge. $E_{xylk}^{NB}$ is similarly defined for negative edges.

|  | $E_{xylk}^{PB}=r\left( E_{xyl}^{P}-c_{k}\sum_{i=1}^{k-1} E_{xy\left( l-i \right)}^{P} \right)$  $E_{xylk}^{NB}=r\left( E_{xyl}^{N}-c_{k}\sum_{i=1}^{k-1} E_{xy\left( l+i \right)}^{P} \right)$ | (43) |
| --- | --- | --- |
|  |  |  |

where $r\left( x \right)$ is a ramp function (21) used in order to assign zero when the blocking edges strength are greater than the blocked edge strength. $T^{E}$ (42) is a normalization factor designed to keep the cost to be per coordinate and orientation:

|  | $T^{E}=\sum_{k=1}^{L-1} e_{k}$ | (44) |
| --- | --- | --- |
|  |  |  |

The weight factor $e_{k}$ (42) is defined according to the graph in Figure 5C to give penalty for edge bending, with greater penalty for concave bends, Section ‎2.4.6. $e_{k}$ is determined by the weight function $e\left( u \right)$, which depends on a continuous angle in the range $\left[ 0,2 \right]$. The value $2$ was taken as the full angle of $2\pi$ for the sake of simplicity. $e\left( u \right)$ by itself is constructed from function $e^{H}\left( u \right)$ defined on half range $\left[ 0,1 \right]$ by mirroring $e^{H}\left( u \right)$ in the $Y$ axis and translating by 1 in the $X$ axis, Figure 5C.

|  | $e\left( u \right)=\left\{ \begin{aligned} e^{H}\left( u \right), &0\leq\alpha\leq1 \\ 1-e^{H}\left( u-1 \right), &1<\alpha\leq2 \end{aligned} \right.$ | (45) |
| --- | --- | --- |
|  |  |  |

$e^{H}\left( u \right)$ has the following constraints on the values and derivatives in order to ensure that it is smooth and yields a smaller cost for convex bends:

| **values** | **derivatives** |
| --- | --- |
| $e^{H}\left( 0 \right)=1$ | ${e^{H}}^{'}\left( 0 \right)=0$ |
| $e^{H}\left( \frac{1}{2} \right)=\frac{1}{4}$ | ${e^{H}}^{'}\left( \frac{1}{2} \right)=-\frac{3}{2}$ |
| $e^{H}\left( 1 \right)=0$ | $e^{H}'\left( 1 \right)=0$ |
|  |  |

One of possible functions $e^{H}\left( u \right)$ satisfying these constraints is

|  | $e^{H}\left( u \right)=\left\{ \begin{matrix} 6u^{3}-6u^{2}+1, & 0\leq u<\frac{1}{2} \\ -2u^{3}+6u^{2}-6u+2, & \frac{1}{2}\leq u\leq1 \end{matrix} \right.$ | (46) |
| --- | --- | --- |
|  |  |  |

The weight coefficient $e_{k}$ is defined by

|  | $e_{k}=e\left( \frac{2k}{L} \right)$ | (47) |
| --- | --- | --- |
|  |  |  |

Note that for a straight continuation ($k=L/2$) the cost is $0$. For a convex right angle bend ($k=L/4$) the cost is $1/4$, while for a concave right angle bend ($k=3/4L$) the cost is higher, $3/4$.

The blocking term in (43) increases for high values of $k$, since more positive values of object edge strengths $E_{xyl}^{P/N}$ are included. In order to decrease the influence of summing many values of $E_{xyl}^{P/N}$, the blocking term is multiplied by a compensation factor $c_{k}$:

|  | $c_{k}=1-\gamma\frac{k-1}{L}$ | (48) |
| --- | --- | --- |
|  |  |  |

where $0<\gamma<1$. This means that for minimum $k=1$ there is no change ($c_{k}=1$) and for increasing $k$ the blocking term (43) is multiplied by a factor less than $1$.

## Finding the local minimum process details

Once the cost functional has been defined, the next problem is to find the border ownership map that minimizes the cost functional. It should be emphasized that the unknown of the problem is the border ownership map, meaning that the solution consists of $I^{X}I^{Y}L$ coordinates of vector $\vec{b}$ (2), for which the cost functional $F\left( \vec{b} \right)$ (1) has a local minimum. The process is performed by iterations, till the minimum is found. At iteration $i$, we find the derivative of cost functional at $\vec{b}^{i}$, Section ‎2.6. The derivative $\vec{D}$ is a $I^{X}\times I^{Y}\times L$ vector pointing in the direction of the greatest increase of $F$. To move towards the minimum of $F$, we need to move in the opposite direction $-\vec{D}$. Let us look at the values of the functional $F$ along the vector $-\vec{D}$, that is at the function $g\left( \alpha\right)=F\left( -\vec{D}\alpha\right)$ with $\alpha\mathbb{\in R}$. Consider the Taylor expansion of this function around it’s minimum. Since the derivative at the minimum is zero, the first order component vanishes, meaning $g\left( \alpha\right)$ around the minimum is roughly second order. Hence the functional $F$ near the minimum is roughly second order (parabolic). Based on this insight, we approximate the values of $F$ along $-\vec{D}$ by a parabola and move to its minimum. Note that this method differs from the classical gradient descent (Curry, 1944) in the approximation by parabola. Our approach proved to perform faster than the classical method. The border ownership maps along $-\vec{D}$ have the form

|  | $\vec{b}^{'}=\vec{b}^{i}-\vec{D}\lambda$ | (49) |
| --- | --- | --- |
|  |  |  |

where $\lambda>0$. The stop condition for the minimization is that the border ownership is sufficiently close to the actual minimum border ownership, thus we require that the border ownership components will be closer than $\eta>0$ to minimum border ownership components. We call $\eta$ the border ownership precision since it defines how close is the border ownership found to the actual minimum border ownership. The problem here is to find $\lambda$ such that the border ownership $\vec{b}^{'}$ will be close to minimum border ownership. To derive the sufficient precision $\delta$ required for $\lambda$, we normalize the border ownership precision $\eta$ by a maximum derivative component.

|  | $\delta=\frac{\eta}{\max_{x,y,l} \left\vert D_{xyl} \right\vert}$ | (50) |
| --- | --- | --- |
|  |  |  |

To make the parabola approximation, the values of $F$ are evaluated for at least 4 points with the following values of $\lambda$: $0,\delta,2\delta,\ldots,2^{j}\delta,\ldots$ with $j=0,1,\ldots$. The cost functional $F$ is evaluated for increasing values of $\lambda$, until $F$ starts to increase or until 10 points are evaluated.

The least square fit method is used to find the parabola $a\lambda^{2}+b\lambda+c$ which is closest to the measured points of the functional $F$. Suppose the $\lambda$ values for which $F$ was evaluated are $\lambda_{i}$ and the values of $F$ are $F_{i}$. We need to find $a$, $b$, $c$ such that the square error

|  | $\sum_{i} \left( a{\lambda_{i}}^{2}+b\lambda_{i}+c-F_{i} \right)^{2}$ | (51) |
| --- | --- | --- |
|  |  |  |

is minimum. It can be easily found by differentiation with respect to $a$, $b$ and $c$ that

|  | $\left[ \begin{matrix} \sum_{i} {\lambda_{i}}^{4} & \sum_{i} {\lambda_{i}}^{3} & \sum_{i} {\lambda_{i}}^{2} \\ \sum_{i} {\lambda_{i}}^{3} & \sum_{i} {\lambda_{i}}^{2} & \sum_{i} \lambda_{i} \\ \sum_{i} {\lambda_{i}}^{2} & \sum_{i} \lambda_{i} & n \end{matrix} \right]\left[ \begin{matrix} a \\ b \\ c \end{matrix} \right]=\left[ \begin{matrix} \sum_{i} F_{i}{\lambda_{i}}^{2} \\ \sum_{i} F_{i}\lambda_{i} \\ \sum_{i} F_{i} \end{matrix} \right]$ | (52) |
| --- | --- | --- |
|  |  |  |

where $n$ is the number of probe points. If $a>0$ then $\lambda$ is set to be $\lambda_{min} =-b/{2a}$ (the parabola minimum). If $\lambda_{min}<\delta$ or if $a\leq0$ then we set $\lambda_{min}=\delta$ to make the smallest progress in the descending direction. Let $\vec{b}^{min}$ be the $\vec{b}'$ value (49) for $\lambda=\lambda_{min}$. If the cost $F\left( \vec{b}^{min} \right)$ is lower than the cost $F\left( \vec{b}^{i} \right)$, $\vec{b}^{i}$ is the border ownership at iteration $i$, the border ownership of next iteration $\vec{b}^{i+1}$ is set to $\vec{b}^{min}$ and the process of finding the derivative and descending to the parabola minimum is repeated for the new border ownership. The minimum with accuracy of $\eta$ is reached when the cost $F\left( \vec{b}^{min} \right)$ is not lower than the cost $F\left( \vec{b}^{i} \right)$. This is the condition to proceed to a lower scale parameter, Section ‎2.5.4, until the desired resolution of minimum scale parameter is reached.

## Repulsive particles cost term

Here is described the cost functional component introducing the “repulsive particles”, Section ‎2.7, that make sure the border ownership does not come close to already obtained minima of the functional (1). Suppose we have $P$ repulsive particles $\vec{b}^{i}$ with $i=1,2,\ldots,P$ and current border ownership $\vec{b}$, which we will also term as a particle. In order to define the distance between particles that reflects their similarity, we require a more complex function than the distance (27) used to determine if the particle found is same as already discovered particle. The reason for this is the following. When $L$ is large, the angle between adjacent border ownership vectors is relatively small. Therefore, when comparing two border ownership maps, it is not sufficient to compare border ownership values in the same direction only, but values in close directions should also be considered. For similar reason, border ownership values at nearby coordinates should also be considered. To solve these issues, we compare border ownership maps after smoothing them in direction and spatial location. This is similar to comparing border ownership maps at a coarse scale. The smoothing of the border ownership map is given by

|  | ${S\left( \vec{b} \right)}_{xyl}=\frac{1}{T^{S}}\sum_{m=-\left( \frac{L}{4}-1 \right)}^{\frac{L}{4}-1} \cos^{2} \left( 2\pi\frac{m}{L} \right)\left( {b_{xy\left( l+m \right)}}^{2}*f_{xy}^{P} \right)$ | (53) |
| --- | --- | --- |
|  |  |  |

where

|  | $T^{S}=\sum_{m=-\left( \frac{L}{4}-1 \right)}^{\frac{L}{4}-1} \cos^{2} \left( 2\pi\frac{m}{L} \right)$ | (54) |
| --- | --- | --- |
|  |  |  |
|  | $f_{xy}^{P}=\frac{1}{2\pi{\sigma^{P}}^{2}}e^{-\frac{x^{2}+y^{2}}{2{\sigma^{P}}^{2}}}$ | (55) |
|  |  |  |

The filter $f_{xy}^{P}$ is responsible for spatial smoothing. Summing over $m$ collects border ownership values from close directions, $m$ is the difference between directions indexes. The multiplication by $\cos^{2} \left( 2\pi\frac{m}{L} \right)$ is aimed to give bigger influence to closer directions. $T^{S}$ is a normalization factor, retaining the border ownership after smoothing in direction in same magnitude as the border ownership before the smoothing. The distance from current particle $\vec{b}$ to particle $i$ is defined by

|  | $D_{i}=\left[ \sum_{x,y} \frac{1}{L}\sum_{l=0}^{L-1} \left( {S\left( \vec{b} \right)}_{xyl}-{S\left( \vec{b}^{i} \right)}_{xyl} \right)^{2} \right]^{1/2}$ | (56) |
| --- | --- | --- |
|  |  |  |

The repulsive cost term, $F^{P}$, depends on this distance

|  | $F^{P}=\sum_{i=1}^{P} \frac{1}{D_{i}}$ | (57) |
| --- | --- | --- |
|  |  |  |

Note, that the cost functional resembles an electrical potential between repelling charged particles, and increases infinitely when two particles approach one another. The term that is added to cost functional (1) is

|  | $\alpha^{P}F^{P}$ | (58) |
| --- | --- | --- |
|  |  |  |

where $\alpha^{P}$ is the repulsive term weight parameter. Note that $\alpha^{P}$ has the same role as the other weight parameters $\alpha^{type}$ in (1). To increase the repulsive force, the repulsive term is multiplied by the repulsive force multiplication factor $\tau$, Section ‎2.7.
